# AI‐Enhanced CAD in Low‐Dose CT: Balancing Accuracy, Efficiency, and Overdiagnosis in Lung Cancer Screening

**DOI:** 10.1111/1759-7714.15499

**Published:** 2024-11-27

**Authors:** Yun‐Ju Wu, Fu‐Zong Wu

**Affiliations:** ^1^ Department of Radiology Kaohsiung Veterans General Hospital Kaohsiung Taiwan; ^2^ Department of Software Engineering and Management National Kaohsiung Normal University Kaohsiung Taiwan

Dear Editor,

We have reviewed the article titled “Application of Artificial Intelligence in Lung Cancer Screening: A Real‐World Study in a Chinese Physical Examination Population,” recently published in *Thoracic Cancer* [1]. This study assessed the diagnostic accuracy of an AI‐assisted system for detecting and differentiating pulmonary nodules compared to manual interpretation by physicians. A cohort of 23 336 patients who underwent low‐dose chest CT screening for lung cancer at West China Hospital was analyzed. The results showed that AI‐assisted readings significantly outperformed manual interpretation, with a higher screening positive rate and an increased likelihood of diagnosing malignant nodules (*p* < 0.001). The AI group demonstrated a superior detection rate for malignant pulmonary nodules (97.2%) compared to the manual group (86.4%, *p* < 0.001), and the lung cancer detection rate was also higher in the AI‐assisted group (98.9% vs. 90.3%, *p* < 0.001). These findings highlight AI's potential as an effective adjunct in lung cancer screening, offering enhanced accuracy and efficiency in identifying malignant nodules [2]. AI systems could alleviate radiologists' workload by improving early detection rates, which is crucial for better patient outcomes [3]. The study supports integrating AI into routine clinical practice to augment traditional screening methods and improve lung cancer detection, particularly in high‐risk populations.

The application of AI in lung cancer screening for the Asian population presents both significant advantages and notable challenges. AI tools can enhance sensitivity and specificity in detecting and classifying lung nodules, especially in a population with a higher incidence of nonsmoking‐related lung cancer [4, 5]. This can reduce false positives and unnecessary follow‐up investigations, which is important for both patient outcomes and healthcare efficiency. AI can also improve the cost‐effectiveness of screening programs, making lung cancer screening more accessible, particularly in countries with limited healthcare resources [6]. This technology allows for more targeted and efficient use of resources, potentially expanding eligibility for screening programs and providing opportunities for incidental findings, such as sarcopenia or cardiovascular diseases, which may improve overall population health [7, 8]. However, there are challenges. AI models risk perpetuating biases if they are not trained on datasets that adequately represent the Asian population, where lung cancer characteristics may differ from Western populations [9, 10]. The concern of overdiagnosis, particularly in populations with a high prevalence of indolent tumors such as ground‐glass nodules (GGNs) or part‐solid nodules (PSNs), poses a risk of unnecessary interventions [11, 12, 13]. This can lead to psychological and financial burdens, causing anxiety and uncertainty in clinical decision‐making. While AI can increase nodule detectability, especially for GGNs, the challenge lies in managing the balance between identifying early‐stage cancers and avoiding overtreatment for indolent tumors that may never pose a significant health risk [14]. In lung cancer screening in Asia, where there is a high prevalence of lung adenocarcinoma among nonsmokers, particularly among women, clinical decision‐making often presents a dilemma due to the heterogeneous growth pattern of PSNs, which can range from rapid to slow [15]. AI tools can enhance the detection of GGNs making them especially useful for screening high‐risk nonsmoking female populations in Asia. However, many detected GGNs are often smaller than 1 cm. Although both the Lung‐RADS and Fleischer guidelines recommend long‐term active surveillance of at least 5 years for such cases, the high detection rate of small GGNs by AI tools may lead to patients seeking early surgical intervention out of fear of lung cancer mortality or seeking second opinions, resulting in over‐surveillance and a waste of medical resources [16, 17].

Therefore, future AI tools should not only focus on detecting GGNs but also integrate clinical data, serial imaging, or genetic information through multi‐omics approaches to identify high‐risk GGNs with aggressive growth patterns [18]. This would further optimize the benefits of lung cancer screening in Asian nonsmokers and reduce the problems of overdiagnosis and overtreatment that are often criticized in lung cancer screening programs in this population.

## Author Contributions

Conceptualization: Designed the research framework and identified key study objectives: Yun‐Ju Wu; Fu‐Zong Wu. Writing ‐ Review and Editing: Critically reviewed and edited the manuscript for intellectual content: Yun‐Ju Wu; Fu‐Zong Wu.

## Conflicts of Interest

The authors declare no conflicts of interest.

## Data Availability

The authors have nothing to report.
